# *Encyclia inopinata* (Orchidaceae, Laeliinae) a new species from Mexico

**DOI:** 10.3897/phytokeys.58.6479

**Published:** 2016-01-12

**Authors:** Carlos L. Leopardi-Verde, German Carnevali, Gustavo A. Romero-González

**Affiliations:** 1 Centro de Investigación Científica de Yucatán, A. C., Calle 43 #130, Colonia Chuburná de Hidalgo, Mérida 97200, Yucatán, México; 2 Current adress: Facultad de Ciencias Biológicas y Agropecuarias, Universidad de Colima, Km. 40 Autopista Colima-Manzanillo. Crucero de Tecomán, Tecomán 28100, Colima, México; 3 Orchid Herbarium of Oakes Ames, Harvard University Herbaria, 22 Divinity Avenue, Cambridge, Massachusetts 02138, U.S.A.

**Keywords:** Encyclia diota, Encyclia insidiosa, Pacific slope, Oaxaca

## Abstract

A new species of *Encyclia* from Mexico, *Encyclia inopinata*, is described and illustrated. This species is similar to *Encyclia diota* but it can be distinguished by its usually more robust plants with 2–3 leaves per pseudobulb and its flowers with longer and narrower sepals (1.8±0.1 × 0.63±0.03 cm in *Encyclia inopinata* versus 1.48 ±0.14 × 0.65±0.06 cm in *Encyclia diota*) and petals (1.7±0.05 × 0.59±0.05 cm in *Encyclia inopinata* vs. 1.36 ±0.19 × 0.81±0.13 cm in *Encyclia diota*), and the labellum with narrower lateral lobes (0.18±0.02 cm in *Encyclia inopinata* vs. 0.41±0.10 cm in *Encyclia diota*). Other characters that differentiate these two species are the coriaceous sepals, pink callus, and white anther of *Encyclia inopinata* (versus fleshy-leathery sepals, white callus, and yellow anther of *Encyclia diota*). The new species can be found in deciduous forests along the Pacific slope of Oaxaca state, near of the border with Guerrero state, at about 1200 m. It blooms between March and July.

## Abstract (es)

Una nueva especie de *Encyclia* de México, *Encyclia inopinata*, es descrita e ilustrada. Esta especie es similar a *Encyclia diota*, pero puede distinguirse por sus plantas usualmente más robustas con 2–3 hojas por pseudobulbo y sus flores con sépalos y pétalos más largos y angostos (sépalos: 1.8±0.1 × 0.63±0.03 cm en *Encyclia inopinata* versus 1.48 ±0.14 × 0.65±0.06 cm en *Encyclia diota*; pétalos: 1.7±0.05 cm de largo en *Encyclia inopinata* vs 1.36 ±0.19 cm en *Encyclia diota*), el labelo tiene lóbulos laterales más angostos (0.18±0.02 cm en *Encyclia inopinata* vs. 0.41±0.10 cm en *Encyclia diota*). Otros caracteres que distinguen a las dos especies son los sépalos coriáceos, callo rosa y antera blanca de *Encyclia inopinata* (versus los sépalos carnoso-coriáceos, callo blanco y antera amarilla de *Encyclia diota*). La nueva especie se encuentra en un bosque deciduo del estado de Oaxaca, en la Vertiente del Pacífico, cerca de los límites con el estado de Guerrero, a 1200 m. Florece entre marzo y julio.

## Introduction

*Encyclia* is a Neotropical genus ranging from Florida in the southern United States of America south to the Salta province in northern Argentina; the distribution includes both the continental areas and the West Indies. Species of the genus are usually found in seasonally dry ecosystems below 1200 m, although some can be found at altitudes of up to 2500 m (van den Berg and Carnevali 2005). According to preliminary phylogenetic analyses, the *Encyclia diota* complex has at least three species, and is morphologically characterized by pyriform pseudobulbs, linear-oblong, relatively wide, and coriaceous leaves, a fractiflex inflorescence with loosely arranged, relatively large, leathery to fleshy-leathery showy flowers, a smooth pedicellate ovary; the sepals and petals are of variable colors, from bronze or ocher to dark chocolate; the labellum is conspicuously trilobulate, the lateral lobes are patent at the apical half or only slightly recurved distally; the callus is glabrous and the column is straight. One of the most distinctive characters of this species complex is the yellow labellum with crimson to reddish brown lines. Members of the *Encyclia diota* complex tends to occupy dry, seasonally deciduous forests, from Mexico (northwestern Oaxaca) to northern Nicaragua, generally at medium to high altitudes (600–2000 m).

Historically, whether *Encyclia diota* represents one or more species has generated considerable disagreement. Some taxonomists propose the existence of at least two species (*Encyclia diota* and *Encyclia insidiosa* (Rchb. f.) Schltr.), a view we support; others suggest the complex consists of one species (consult Dressler 1976, for a historical review). These discrepancies are reasonable because, on the one hand, plants from different populations are, at first, difficult to distinguish from each other but, on the other hand, careful examination can show clear and consistent morphological differences between populations, strongly associated with a geographical component, a biological scenario that may require more than one species to be understood (Leopardi-Verde et al. in prep.).

We recently detected yet another species in this complex that shows differences in shape, size, and colors from others in the complex, which is here described and illustrated.

## Materials and methods

Live plants were studied ex situ, from collections of German Carnevali and the Missouri Botanical Garden. We also examined 49 specimens housed at herbaria AMES, AMO, CICY, F, MEXU, MO, TEFH and W. Of these, 23 were of *Encyclia diota* and 21 of *Encyclia insidiosa*. Acronyms of the herbaria follows Thiers (2013). The characters (vegetative and floral) used to describe the new species were determined from the fresh and dry specimens. In case of fresh materials and dry specimens, pictures of whole samples were taken with a FUJIFILM FinePix S200EXR digital camera. Fresh flowers were dissected and digitized in a XL1600 EPSON scanner. For rehydratation, herborized flowers were placed in a commercial detergent with ammonia for about 30–45 min, and then left in water for about one hour; afterwards they were dissected and mounted in a glass slide to digitize in a XL1600 EPSON scanner. Each specimen or its details were digitized with a scale. The cardboard file mounted records were scanned directly. All measures (height, length and width of pseudobulb, leaves, length of the inflorescence and its branches, etc.) were taken with the software ImageJ (Schneider 2012).

## Data resources

The data underpinning the analysis reported in this paper are deposited at GBIF , the Global Biodiversity Information Facility, http://ipt.pensoft.net/resource.do?r=encyclia_diota_complex.

## Taxonomy

### Encyclia inopinata

Taxon classificationPlantaeAsparagalesOrchidaceae

Leopardi, Carnevali & G.A.Romero sp. nov.

urn:lsid:ipni.org:names:77151882-1

Figures 1 , 2

#### Type.

MEXICO. **Oaxaca**: Distrito de Tlaxiaco, Municipio de Santiago de Yosondúa, 2.8 km. al SSE en línea recta (4-5 mm. por carretera) de Santiago de Yosondúa por la vía a Yerba Santa, 16°49'18.26"N, 97°35'11.63"W, 1267 m, 24/VI/2010, *G. Carnevali & C. Leopardi 7139* (Holotype CICY; isotypes AMES, AMO).

#### Diagnosis.

*Encyclia inopinata* is similar to *Encyclia diota*, but it can be distinguished by its flowers with longer and narrower sepals (1.8±0.1 × 0.63±0.03 cm in *Encyclia inopinata* versus 1.48±0.14 × 0.65±0.06 cm in *Encyclia diota*) and petals (1.7±0.05 × 0.59±0.05 cm in *Encyclia inopinata* vs. 1.36±0.19 × 0.81±0.13 cm in *Encyclia diota*), and the labellum with narrow lateral lobes (0.18±0.02 in *Encyclia inopinata* vs. 0.41±0.10 cm in *Encyclia diota*). Other characters that differentiate these two species are the coriaceous sepals, pink callus, and white anther of *Encyclia inopinata* (versus fleshy-leathery sepals, white callus, and yellow anther of *Encyclia diota*).

#### Description.

**Figure 1.**
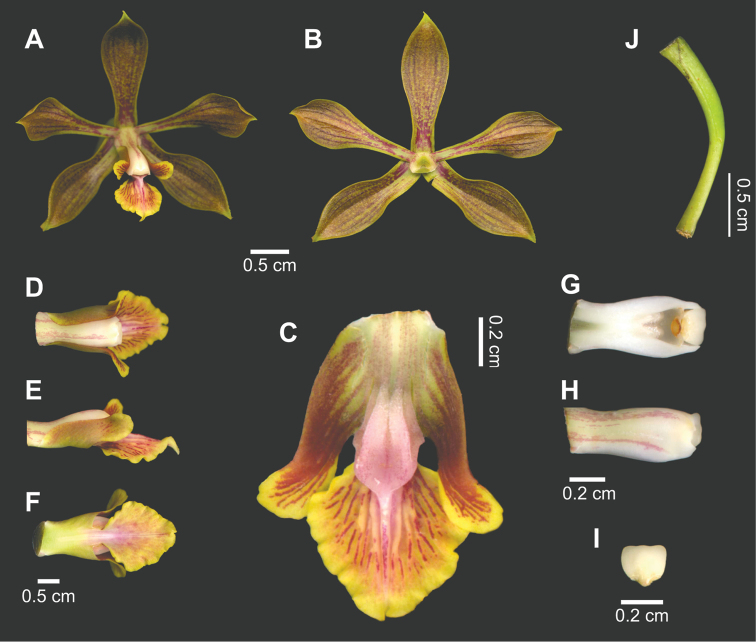
*Encyclia inopinata*. **A** Flower in frontal view **B** Perianth **C** Spread labellum **D−F** Column and labellum, in natural position, in dorsal (**D**) lateral (**E**) and ventral (**F**) views **G–H** Column in ventral (**G**) and lateral (**H**) views **I** Anther **J** Pedicellate ovary. Based on the holotype.

Epiphytic *herb*, 30–42 cm tall, up to 80–90 cm including the inflorescence. Rhizome short and fibrous. *Pseudobulbs* 5.0–8.0 × 3.9–4.5 cm, clustered, ovoid to pyriform, apically 2–3-leaved, green and smooth when young, covered with papery sheaths that eventually defibrate and disintegrate, when old sometimes stained with maroon or purple. *Leaves* 34–38 × 2.8–3.5 cm, linear-oblong to oblong-ligulate, subacute, coriaceous, conduplicate at the base, dark green to purple tinged with central nerve marked mainly on abaxial face. *Inflorescence* 60–90 cm long, terminal, erect, racemose or paniculated, when panicles with 3–5 branches of 2.6–11 cm long, each branch with 3–8 flowers, the entire inflorescence with up to 50 flowers; peduncle slender but strong, smooth, usually green, with adppressed sheaths of 0.9–1.4 cm long, that become smaller toward the apex; bracts inconspicuous, triangular of 0.2–0.5 cm long; pedicellate ovary 1.3–1.7 cm long, smooth. *Flowers* resupinate, showy, 3.0–3.4 cm diameter (between the tips of the petals); sepals and petals coriaceous, bronze-green, veins marked with dark purple lines; labellum green towards the base and pale, dull yellow-green toward the apex, with reddish-brown lines, the central lobe with well developed keels that reach the apex, of these the most conspicuous is the central one, lateral lobes with reddish brown lines that extend almost to the apex, lateral lobes free of the central lobe, pale pink callus; column creamy white with reddish-brown spots and lines; sepals similar, oblanceolate, acute, the laterals oblique, dorsal sepal 1.7–1.9 × 0.60–0.65 cm, lateral sepals 1.7–1.9 × 0.60–0.65 cm; petals 1.65–1.73 × 0.57–0.60 cm, obovate-spatulate to narrowly obovate-spatulate, with a conspicuous claw towards the base of 0.64–0.68 × 0.10–0.16 cm, acute to acuminate. *Labellum* 1.25–1.35 × 0.9–1.1 cm, 3-lobulate, free of the column except at the base, central lobe 0.5–0.7 × 0.6–0.8 cm, ovate, shortly acuminate; lateral lobes 0.80–0.90 cm long, 0.17–0.20 cm wide in the middle portion and 0.4–0.5 cm wide in the portion that lies between the base of the labellum and which separates within the central lobe, oblong, rounded towards the apex, separated of the central lobe by a sinus of ca. 0.1 cm width; in natural position lateral lobes are reflexed at the apex, and embrace the column; callus 0.45–0.49 × 0.24–0.28 cm, sub-rhombic, hirsute, consisting of two keels separated by a conspicuous sulcus, which widens slightly and forms a fovea, these keels converge towards the apex of the callus extending into the blade of the central lobe of the labellum as a keel that reaches the apex. *Column* 0.6–0.7 × 0.2–0.3 cm, semi-cylindrical, straight, ventral outline slightly clavate, wingless; anther 0.18–0.22 × 0.18–0.22 cm, white; pollinia 4, yellow, separated into groups of two, yellow caudicles; stigmatic surface 0.23–0.29 × 0.19–0.24 cm, subquadrate; rostellum 0.060–0.064 × 0.149–0.153 cm, semicircular. *Capsule* not seen (Figure 1 A–J).

#### Additional specimens examined.

MEXICO. **Oaxaca**: El Llano, IV/1964, *Miller* sub *G. Pollard E-34* (AMO; card); km. 230 Puerto Escondido Highway to Oaxaca, V/1964, *G. Pollard E-45* (AMO; card); km. 154 Puerto Escondido Highway, 28/I/1969, *G. Pollard E-209-D* (AMO; card); Municipio San Juan Cacahuatepec, ca. 10.5 km north of San Juan Cacahuatepec, 15/III/1967, *G. Pollard E-209-C* (AMO; card).

#### Distribution and ecology.

**Figure 2.**
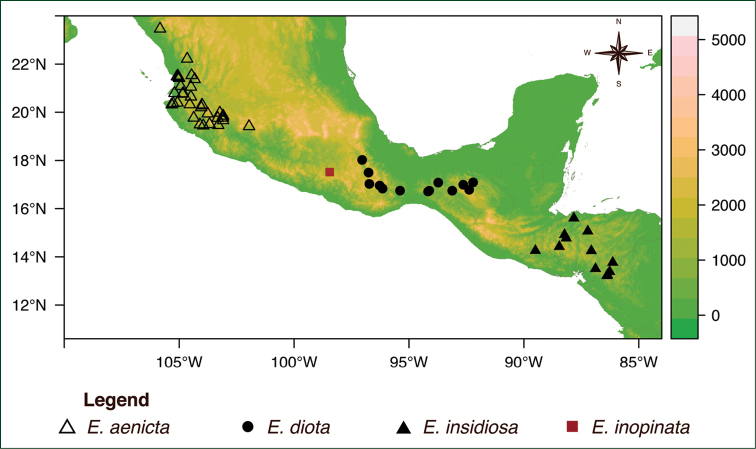
Distribution of species of the *Encyclia diota* complex. The color bar in the right side represents the altitude in meters.

*Encyclia inopinata* has been reported only from Oaxaca state near the pacific slope from north of Puerto Escondido to north of San Juan Cacauatepec (near the border with the Guerrero state) in deciduous forest, ca. 1200 m (Figure 2). It usually grows as a lithophyte. This species appears to be rare. It blooms between March and July.

#### Etymology.

From the Latin *inopinatus*, unexpected, in reference to the surprise that we felt when first seeing the new species in bloom and realizing that it was an undescribed species in the *Encyclia diota* complex.

#### Comments.

Dressler and Pollard (1974: 130) suggested that a population of *Encyclia aenicta* in Oaxaca “tend[s] to have longer and wider lateral lobes and also tends to be more yellowish in color, suggesting, some hybridization with *Encyclia diota*.” These authors implied that, size-wise, this entity was more similar to *Encyclia aenicta* than to *Encyclia diota*. The locality and characters showed by Dressler and Pollard (1974: 130) strongly suggests that they were referring to a population of what is here proposed as *Encyclia inopinata*. Another source containing information about an entity similar to *Encyclia inopinata* is the “Notes on *Encyclia*”, and unpublished manuscript of G. Pollard (housed at the AMO library). Tome 4 (“N to O”), page 69, describes a specimen that matches well our concept of *Encyclia inopinata*. This manuscript also has additional cards (E-34 y E-45, p. 70 and 72 respectively) and pictures (p. 71) showing specimens that, again, match well the new species proposed herein.

Hybridization, most likely, has played an important role in the evolution and diversification of *Encyclia* (Dressler and Pollard 1974, Leopardi-Verde 2014). However, in this case, it is difficult to support the hypothesis of a cross between *Encyclia diota* and *Encyclia aenicta* as the origin of *Encyclia inopinata* (Dressler and Pollard 1974: 130). We strongly reject this hypothesis considering that there is no contact zone between these taxa (Figure 2) and, in addition, the lack of morphological intermediacy between the hypothetical parents of such hybrid.

Finally, *Encyclia inopinata* resembles *Encyclia diota* in floral colors, but the texture and proportions of the flowers are very different (see diagnosis above). *Encyclia inopinata* is also similar to *Encyclia insidiosa*, an entity from Central America (ranges from Nicaragua to northern Guatemala). *Encyclia inopinata* and *Encyclia insidiosa* can be discriminated by the tendency of the second to have sepals and petals dirty ocher to chocolate, whereas in *Encyclia inopinata* these structures are bronze-green. The anther in *Encyclia insidiosa* is yellow whereas in *Encyclia inopinata* it is white or creamy white. The sepals and petals in *Encyclia insidiosa* are shorter than in *Encyclia inopinata* (1.54–1.68 cm versus 1.7–1.9 cm). The labellum of *Encyclia insidiosa* and *Encyclia inopinata* are similar in length, but in the former is wider (1.22–1.38 × 1.31–1.61 cm versus 1.25–1.35 × vs. 0.9–1.1 cm).

### Key to the species of the *Encyclia diota* complex

**Table:** 

1	Sepals and petals fleshy-leathery, ocher, broadly spatulate, usually rounded or sub-squared towards the apex. Petals less than 1.5 cm long and more than 0.8 cm wide (ratio length/width in petals less than 1.9). Lateral lobes of the labellum ca. 0.9±0.12 cm long and 0.4±0.10 cm wide in the apical portion	***Encyclia diota*** (Figure 3)
–	Sepals and petals leathery or coriaceus, brown or bronze, obovate-spatulate to narrowly-obovate-spatulate, usually acute towards the apex. Petals more than 1.6 cm long and less than 0.7 cm wide (ratio length/width in petals more than 2.0). Lateral lobes of the labellum with ca. 0.8±0.06 cm long or less and 0.3±0.05 cm width in the apical portion	**2**
2	Plants of Central America. Petals 1.6 cm long, rare 1.7 cm (ration length/width: 2.5 or less), dirty ocher to chocolate. Labellum more than 1.3 cm wide (ratio length/width: less than 0.95). Callus white. Anther yellow	***Encyclia insidiosa*** (Figure 4)
–	Plants of Oaxaca (Mexico). Petals 1.8 cm long or more (ration length/width: 2.8 or more), bronze-green. Labellum less than 1.1 cm wide (ratio length/width: more than 1.2). Callus pink. Anther white or creamy white	***Encyclia inopinata*** (Figure 1)

**Figure 3.**
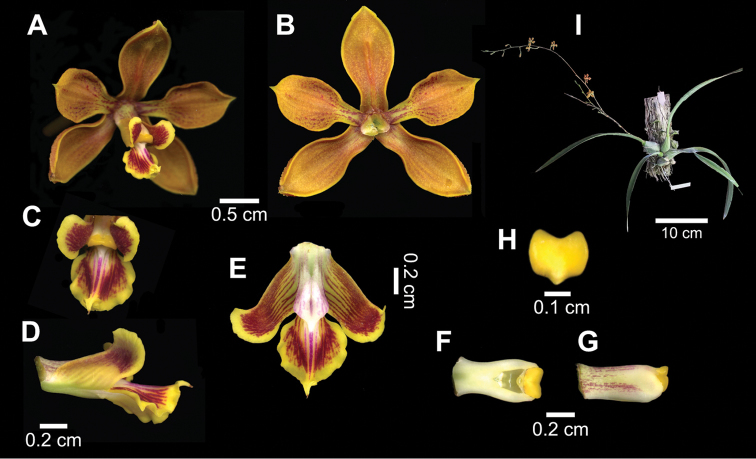
*Encyclia diota*. **A** Flower in frontal view **B** Perianth **D−F** Column and labellum, in natural position, in frontal (**C**) and lateral (**D**) views **E** Spread labellum **F–G** Column in ventral (**F**) and lateral (**G**) views. H, Anther. I, Plant. Based on *Leopardi 337* (CICY).

**Figure 4.**
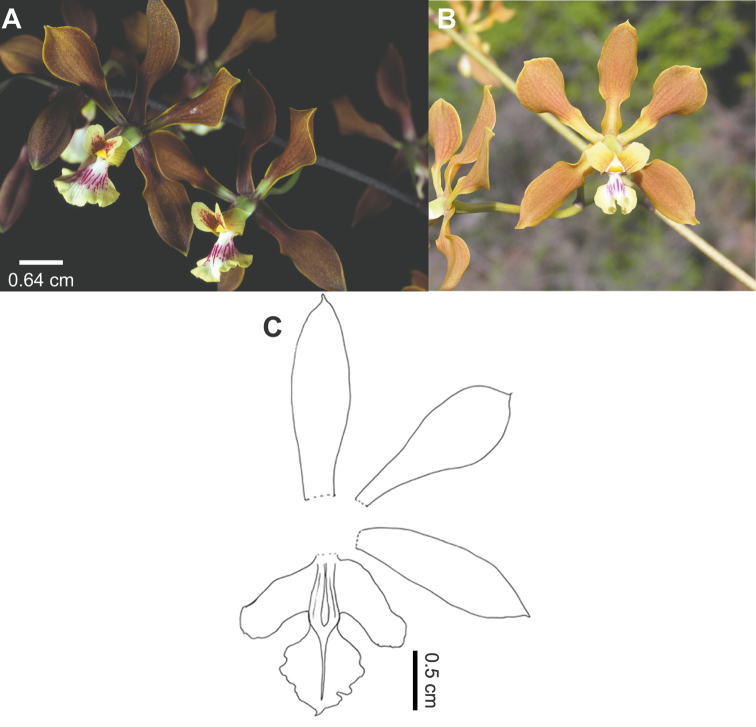
*Encyclia insidiosa*. **A−B** Flower in frontal view of different phenotypes **A** from Nicaragua **B** from Honduras **C** Dissection of a flower **A** based on Stevens 20535 (MO), photo O. Montiel **B** based on House s.n., photo P. House **C** Drawn by C. Leopardi from a re-hydrated flower with the aid of a lucid camera, based on *Edwards 410* (AMES).

## Supplementary Material

XML Treatment for Encyclia inopinata
